# Air caloric test reference values

**DOI:** 10.1590/S1808-86942012000300001

**Published:** 2015-10-14

**Authors:** Sérgio Albertino, Roseli Saraiva Moreira Bittar, Marco Aurelio Bottino, Maurício Malavasi Ganança, Denise Utsch Gonçalves, Mario Edvin Greters, Raquel Mezzalira, Fernando Freitas Ganança

**Affiliations:** ABORL-CCF – Department of Otoneurology

Electronystagmography (ENG) and videonystagmography (VNG) are important tools in otoneurological diagnosis and are widely used in Brazil. The caloric test, included in the set of ENG and VNG evaluations, is the only test that analyzes each labyrinth separately. It consists of hot or cold stimulation of the external ear canal, intending to warm up or cool down the wall of the lateral semicircular canal causing endolymph convection currents. These currents cause further deviation from the dome of the stimulated semicircular canal. Heat stimuli are used worldwide in the routine workup of the vestibular system and can be performed with water or air.

In Brazil, we face the problem of the large variability in the values of the slow component speed of nystagmus in air-stimulated ENG or VNG, which follows standards from each clinic, not always in compliance with international standards. In order to establish a reference, the Department of Otoneurology of the Brazilian Association of Otorhinolaryngology and Neck and Facial Surgery - ABORL-CCF - decided to study the response to an air caloric test in healthy individuals in different age groups with the aim of providing colleagues with information regarding international standards of air stimulation (24 º and 50 º C) for the Brazilian population. After publication, the values will be adopted by members of the Department of Otoneurology and their respective clinics, as a criterion for identifying vestibular dysfunction upon ENG or VNG.

The study of the caloric test with air at 24 ° and 50 °C, during 1 minute, with air flow of 8 liters / minute, using the air stimulation equipment available in our country, was held at USP, UNIFESP, UFMG and UNICAMP in healthy individuals, males and females, aged between eight and 80 years. There was no spontaneous nystagmus in our sample of healthy individuals. The post-caloric nystagmus Slow Component Angular Velocity (SCAV) minimum value was 3º/s and the maximum was 46º/s. The results of 211 caloric tests with air at 24° and 50°C, alternately in each ear (total of four stimulations in each healthy individual) were subjected to statistical analysis.

Based on statistical evaluation of the results, the values to be considered to characterize vestibular dysfunctions upon the caloric test with ENG or VNG are the following:

## Relative values

• Labyrinthine predominance (PL) > 19%
• Directional Preponderance (DP) > 17%

## Absolute values

• Hyporeflexia
  Unilateral: Sum the SCAV of values of the cold and hot tests in the right and left ears: <5º/s. Bilateral: Sum of the SCAV in the four tests <12º/s.
• Hyperreflexia
  Unilateral: Sum of the SCAV in the cold and hot tests in the right and left ears: > 62º/ s Bilateral: Sum of the SCAV in four tests > 122º/s

*Note: a caloric test without abnormalities does not rule out vestibular dysfunction; the findings in this test must be interpreted together with the patient's clinical evaluation.*

